# Theaflavin Attenuates TBHP-Induced Endothelial Cells Oxidative Stress by Activating PI3K/AKT/Nrf2 and Accelerates Wound Healing in Rats

**DOI:** 10.3389/fbioe.2022.830574

**Published:** 2022-03-02

**Authors:** Dalei Chen, Zhijian Wu, Lu-Ning Wu, Jingtao Jiang, Gui-Nv Hu

**Affiliations:** ^1^ Department of Thyroid and Breast Surgery, Affiliated Dongyang Hospital of Wenzhou Medical University, Dongyang, China; ^2^ Department of Orthopaedics, The Second Affiliated Hospital and Yuying Children’s Hospital of Wenzhou Medical University, Wenzhou, China

**Keywords:** Theaflavin, PI3K/AKT/Nrf2 signaling pathway, oxidative stress, angiogenesis, wound healing

## Abstract

The treatment of wounds remains a clinical challenge because of poor angiogenesis under the wound bed, and increasingly, the patients’ need for functional and aesthetically pleasing scars. Previous reports have shown that Theaflavin can induce angiogenesis and terminate the progression of ischemic cardiovascular disease, but limited therapy is available for the management of cutaneous wounds. In this study, our *in vitro* work discovered that human umbilical vein endothelial cells (HUVECs) exposed to Theaflavin can alleviate apoptosis and cell dysfunction induced by tert-butyl hydroperoxide (TBHP). The cellular activity of HUVECs were assessed by cell tube formation, migration and adhesion. Mechanistically, Theaflavin protected HUVECs from TBHP-stimulated cell apoptosis through the activation of the phosphatidylinositol-3-kinase (PI3K)/protein kinase B (AKT)/nuclear factor (erythroid-derived 2)-like 2 (Nrf2) axis, so Nrf2 silencing can partly eliminate the cytoprotective effect of Theaflavin treatment. In *in vivo* experiments, administering Theaflavin orally can enhance vascularization in regenerated tissues and accelerate wound healing. In summary, our data served as a novel evidence for the wound healing treatment with Theaflavin, and certified the potential mechanism of Theaflavin, which can be used as a potential agent for cutaneous wound therapy.

## Introduction

The skin can block the body from direct external stimulation, so it is regarded as the most susceptible damaged tissue (Sen et al., 2016). Skin wounds can be induced by mechanical, chemical, electrical and thermal wounds or persistent illnesses. Skin wounds pose a widespread global threat to the quality of life and socio-economic conditions (Boer et al., 2016; Gallo, 2017). These wounds not only cause pain, but also become infectious, requiring amputations among a large group of patients worldwide. Therefore, it is of great clinical significance to take novel strategies to accelerate the healing process of wound.

Wound healing is a complicated procedure that encompasses the collaboration between keratinocytes, fibroblasts, endothelial cells, macrophages, and platelets (Broughton et al., 2006; Velnar et al., 2009). Angiogenesis is thought to play a substantial role during the healing of wounds, which is primarily done by endothelial cells (Tonnesen et al., 2000; Veith et al., 2019). During wound healing, the microvasculature axis containing angiogenic capillaries can provide regenerated tissues with nutrients and oxygen (Davidson and Mustoe, 2001; Tejada et al., 2019). But when oxygen is used to generate energy through oxidative phosphorylation, reactive oxygen species (ROS) are synthesized, thereby increasing oxidative stress at the wound site and causing additional endothelial damage (Liu et al., 2019). Hence, it is probably a potential therapeutic target for protecting endothelial cells from damage and improving wound healing.

Heme oxygenase (HO)1 is activated under the condition of oxidative stress. It is a key molecule for the abrogation of oxidative stress (Zhang H. et al., 2018; Xiao et al., 2018). In addition, in the absence of HO1, endothelial cells become highly susceptible to stress-related injury. Nevertheless, the stress-related injury can be alleviated by activating HO1 (Kinderlerer et al., 2009; Böckmann and Hinz, 2020). The upregulation of HO1 is related to the transcriptional agent nuclear factor-erythroid 2 associated factor 2 (Nrf2) (Sudan et al., 2019; Dong et al., 2020). The activated Nrf2 becomes nuclear-bound where it binds to the antioxidant response element, so as to promote balance in the ratio of oxidants/antioxidants after oxidative stress damage (Ma, 2013; Shaw and Chattopadhyay, 2020). Therefore, we hypothesized that stimulation of the Nrf2/HO-1 signaling axis was probably a protective treatment for endothelial cells against oxidative stress-driven destruction.

In recent years, a variety of natural products possessing antioxidant effects have been attracting significant attention as therapeutic agents for the prevention of oxidative stress-related diseases (Tang et al., 2021; Xu et al., 2021). Among them, theaflavin (TF), a principal constituent of black tea, owns multiple health benefits and is well known for its antioxidant properties owing to its effect on superoxide anion scavenging (Ilacqua et al., 2017; Li et al., 2021). A recent study has clarified that TF ameliorates ionizing radiation-induced hematopoietic stem cell damage mainly by decreasing oxidative stress through activating the Nrf2/HO-1 pathway (Han et al., 2017). It has also been found that TF could attenuate cerebral ischemia/reperfusion injury by abolishing Nrf2 inhibition and reducing oxidative stress (Li et al., 2019). However, their role in wound healing therapy remains unknown.

Previous research has reported that growth factors such as bFGF, PDGF, and VEGF could promote angiogenesis, and several signal proteins including phosphatidylinositol-3-kinase (PI3K), protein kinase B (AkT) and mTOR participate in the wound healing process (Gupta and Qin, 2003; Muñoz-Chápuli et al., 2004; Zhang R. et al., 2018). Interestingly, a research study confirmed that activation of PI3K and AKT can promote the cells proliferation, migration, differentiation, angiogenesis (Wang et al., 2021). However, the potential angio-modulatory and wound healing roles of TF remain poorly understood.

Consequently, in this study, we assumed that TF could promote angiogenesis and accelerate wound healing through activating the PI3K/AKT/Nrf2 signaling pathway. The proangiogenic effect and the underlying mechanism of TF were investigated *in vitro*.

## Methods

### Reagents and Antibodies

Theaflavin (HY-N0243, purity:99.69%) came from MedChemExpress (Monmouth Junction, NJ, United States). Dimethyl sulfoxide (DMSO) and tert-butyl hydroperoxide (TBHP) were obtained from Sigma-Aldrich (St. Louis, MO, United States). C-caspase3 (ab32351), Bax (ab32503), Bcl-2 (ab182858), Cytochrome C (ab133504) antibodies were from Abcam (Cambridge, United Kingdom); Primary antibodies against Nrf2 (16396-1-AP), HO-1 (27282-1-AP), GAPDH (60004-1-Ig), CD31 (11265-1-AP), VEGF (19003-1-AP) and Lamin B (12987-1-AP). These primary antibodies were from Proteintech Group (Chicago, IL, United States), and 4’,6-diamidino-2- phenylindole (DAPI) came from Beyotime (Shanghai, China). All cell culture reagents came from Gibco (Grand Island, NY, United States).

### Cell Culture and Treatment Protocols

HUVECs (ATCC, Manassas, VA, United States) were grown in DMEM/F12 ((Gibco, Invitrogen, Grand Island, NY)) containing 10% thermal-inactivated FBS and 1% penicillin and streptomycin in an incubator at 37°C with 5% CO_2_. Briefly, fresh cells were seeded at a density of 2,500–3,000 cells/cm^2^ in T-75 flasks (Falcon). The medium was changed every 48 h. By light microscopic examination we observed that cultures reached confluence after 6–7 days.

According to previous group experiments (Carracedo et al., 2012), cells passaged 2–9 times were defined as young, cells passaged 15–25 times were defined as intermediate and those passaged >30 times were considered as senescent.

Each huvec type (young, intermediate or senescent) was stimulated for 24 h with different concentrations of Theaflavin (0, 25 and 50 µM) with or without TBHP (500 µM) to determine the apoptosis, oxidative stress and cell proliferation. All assays were quantified and analyzed by flow cytometry on a FACSCalibur cytometer (Becton Dickinson Biosciences (BD); San Jose, CA, United States) equipped with standard CellQuest software. Data were collected for 10,000 cells per sample.

### Cell Viability Assay

Cell Counting Kit-8 (CCK-8) assay (MedChemExpress LLC; Monmouth Junction, NJ, United States) was used to determine cell viability. TBHP can stably cause oxidative stress in endothelial cells, thus it was used as an *in-vitro* stimulus to simulate the oxidative stress process in the survival of wound healing (Jiang et al., 2021). Before being co-treated with Theaflavin (0, 25 and 50 µM) with or without TBHP (500 µM), second-generation HUVECs (1×10^4^ cells/well) were grown on a 96-well plate and incubated in DMEM/F12 medium at 37°C. 24 h later, the cells were rinsed with PBS and then DMEM/F12 serum-free culture containing 10 μL CCK8 was added to each well to incubate for another 2 h. Eventually, the absorbance of cells at 450 nm in each well was recorded by a microwell Plate reader (Thermo Fisher).

### Western Blotting Assay

Western blotting assay was performed as per the conventional protocol (Gu et al., 2021). HUVECs were lysed in 1 mM PMSF (phenylmethanesulfonyl fluoride) radioimmunoprecipitation analysis buffer to extract protein and then quantified with BCA protein assay kit (Beyotime). By following the manufacturer’s guidelines, we used a commercial kit to isolate the nuclear and cytoplasmic protein fractions. Equal amount of protein was isolated by sodium dodecyl sulfate-polyacrylamide gel electrophoresis, transferred onto a polyvinylidene fluoride membrane (BioRad, United States), and then blocked with 5% skim milk at room temperature for 2 h, followed by exposure to the following primary antibodies at 4°C overnight: Cleaved-caspase3 (C-caspase3) (1:1000), Bax (1:1000), Bcl-2 (1:1000), Cytochrome C (1:1000), Nrf2 (1:1000), HO1 (1:1000), or Lamin B (1:800). After that, they were exposed to corresponding HRP-conjugated secondary antibodies for 2 h at room temperature. The washed bands were visualized by using a chemical XRS + imaging system (Biolard, United States) and Image LabV 3.0 (Bio-Rad, United States) was used to quantify protein expression.

### Tunel Assay

*In Situ* Cell Death Detection Kit (Roche, SouthSanFrancisco, CA, United States) was used to measure apoptotic cells, according to the manufacturer’s guidelines. To put simply, the treated HUVECs (1×10^4^ cells/mL) were rinsed with PBS for three times and then fixed in paraformaldehyde (4%) for 20 min at room temperature. Later, the cells were exposed to 3% hydrogen peroxide and freshly-made 0.1% Triton X-100 for 10 min. For each step, the HUVECs were rinsed with PBS for three times and then exposed to TUNEL reagent. We quantified the apoptotic cells in 3 separate arbitrary fields under a fluorescence microscope (Olympus Inc., Tokyo, Japan).

### EdU Staining Assay

According to the manufacturer’s steps, huvec proliferation was assessed by using the intake of 5-acetyl-2′-deoxyuridine (EDU) in DNA with the Clickit EdU Microplate Test Kit (Invitrogen). The treated HUVECs (1 × 10^4^ cells/mL) in each group were incubated with Oregon Green azide conjugated EdU. And then the cells were permeated and reacted with HRP-conjugated anti-Oregon green antibody and Amplex infrared. The stained HUVECs were observed under a fluorescence microscope (Olympus Inc., Tokyo, Japan).

### Mitochondrial Function Determination

HUVECs (1×10^4^ cells/mL) were treated with different concentration TF (0, 25 and 50 μM) for 24 h with or without 500 μM TBHP for 2 h. To detect the changes in mitochondrial membrane potential (MMP), the treated HUVECs were incubated in JC-1 solution (T3168, Invitrogen) at a concentration of 10 mg/L for 20 min at 37°C and then washed 3 times with PBS to remove excess JC-1 solution. Then, the stained samples images were immediately captured under a confocal microscope (Olympus, Tokyo, Japan). Lastly, the relative MMP was analysed with the ImageJ software. The relative MMP was calculated as a ratio of the mean fluorescence intensity of red fluorescence (excitation wavelength, 525 nm; emission wavelength, 590 nm) to green fluorescence (excitation wavelength, 490 nm; emission wavelength, 530 nm). To detect the changes in mitochondrial ROS generation, the treated HUVECs were stained in 5 nM MitoSOX Red Mitochondrial Superoxide Indicator solution (M36008, Invitrogen) for 30 min at 37°C. The fluorescence images were captured via a microscope (Olympus, Tokyo, Japan) under 510 nm excitation wavelength and 580 nm emission wavelength. Quantitation of mean fluorescence intensity by ImageJ was used to compare the ROS changes of the mitochondria.

### Tube Formation Assay

Huvec tube formation was performed on a chamber glass slide coating by means of matrix coagulation. An ECMatrix gel solution was applied on the u-slide plate, and then placed it in a 37°C incubator for 1 h to solidify the matrix. The pretreated HUVECs (1 × 10^4^ cells/mL) were harvested by using trypsin/ethylenediaminetetraacetic acid. The treated HUVECs were plated on the Matrigel processed earlier, and then incubated at 37°C for 6 h. The tube formation was observed under a phase contrast microscope (40 × ) and then estimated by quantifying the selected area of each well randomly.

### Cell Migration Assay

Huvec migration was measured on an 8-μm-pore polycarbonate membrane Boyden chamber insert within a transwell system (Costar, Cambridge, MA, United States). The cells were exposed to Theaflavin (TF) and TBHP as previously described. After that, the cells were separated, centrifuged, and re-suspended. 1 × 10^4^ cells were placed in 200 μL of non-FBS DMEM/F12 medium inside a transwell device, and then 700 μL of medium with 1% FBS was introduced to the bottom chamber. After being placed in a 5% CO_2_ incubator for 12h, the membrane was rinsed with PBS for three times, and then fixed with 4% paraformaldehyde. The transwell apparatus were stained with crystal violet, and cells on the upper surface were removed with swabs. Those that moved to the lower surface were quantified in 3 arbitrary fields (40×).

### Cell-Matrix Adhesion Assay

An huvec adhesion assay was performed on a 6-well plate. HUVECs were exposed to TBHP and Theaflavin (TF) as previously described. The plate was pre-coated with fibronectin (5 μg/ml) at 37°C for 1 h. Equal numbers of harvested cells (1 × 10^4^ cells/mL) were grown on each coated plate and placed in the incubator for 30 min. Later on, the non-adherent cells were rinsed with PBS and then fixed with 4% paraformaldehyde. The adherent cells were detected by DAPI staining. The quantity of cell adhesion was determined in three separate representative areas of each well.

### Small Interfering Ribonucleic Acid Incorporation

To silence human Nrf2 gene (RiboBio, Guangzhou, China), double-stranded siRNA was designed and synthesized, with the following sequence: sense strand *5′-GGT​TGA​GAC​TAC​CAT​GGT​T-3’*. According to the manufacturer’s guidelines, the cells (1×10^4^ cells/mL) were mixed with 50 nM siRNA and Lipofectamine 2000 Reagent (Thermo Fisher, UT, United States) for 36 h, exposed to TF and TBHP as previously described, and then subjected to Western blot analysis.

### Real-Time PCR

TRIzol (Invitrogen) was used to isolate the total celluar RNA from treated HUVECs according to the methods provided by the manufacturer. After that, 1 µg of total RNA in each group was reverse-transcribed with the cDNA synthesis kit (MBI Fermantas, Germany). The PrimeScript-RT reagent kit (TAKARA, Japan) and SYBR Premix Ex Taq (TAKARA) in a CFX96 Real-Time PCR System (Bio-Rad Laboratories, CA, United States) were used to analyze the quantitation of PCR. Glyceraldehyde 3-phosphate dehydrogenase (GAPDH) expression was taken as an internal control by using the 2^−ΔΔCt^ method, to evaluate the expression of target gene. The primer sequences were designed in the laboratory and synthesized by TsingKe Biotech based on the mRNA sequences obtained from the NCBI database as follows: *HO-1, 5′-AGA​GTT​TCT​TCG​CCA​GAG​G-3’ (forward), and 5′-GAG​TGT​GAG​GAC​CCA​TCG-3’ (reverse).*

### Animals and Ethics Statement

Fifty-four healthy male Sprague-Dawley rats (250–300 g) were purchased from Wenzhou Medical University (license no. SYXK [ZJ] 2020-0014) and caged under routine environment (temperature: 23 ± 2°C, humidity: 50 ± 5%, 12 h light/dark cycle). All animal surgical interventions followed the Guide for Care and Use of Laboratory Animals of the China National Institutes of Health. The experiments involving animals received approval from the Wenzhou Medical University’s Animal Research Committee (wydw 2021-0256). For this study, rats were arbitrarily separated into two groups: Control (*n* = 18), TF (*n* = 18).

### Skin Wounding Model Establishment and Drug Administration

2% (w/v) pentobarbital (40 mg/kg) was administered intraperitoneally to anesthetize the rats, and the vital signs of the rats were observed closely during the re-anesthesia. After shaving and disinfecting, two circular wounds with a diameter of 2 cm were made on both sides of the dorsal torso using surgical scissors. To determine the appropriate oral dose of TF, thirty rats were randomly divided into the five groups after surgery (n = 6) and were received TF orally for seven consecutive days with doses of 0, 10, 20, 30, 40 mg/kg, respectively. After the determination of the appropriate TF dose, animals were randomly divided into two groups. The TF group was administered TF (20 mg/kg) intragastrically each day until the day of sacrifice. The control group was treated with same dose of normal saline. On day 7, day 14, and day 21 after wounding, the rats were euthanized with excessive pentobarbital sodium, and the wounds and adjacent tissues were sampled for histological evaluation.

### Laser Doppler Blood Flow Imaging

For the purpose of visualizing circulation under the flap, the rats were anesthetized, made to lie prone and then measured with a laser doppler instrument (Moor Instruments, Axminster, United Kingdom) day 7, day 14, and day 21 after surgery. Moor LDI View (ver.6.2; Moor Instruments) was adopted to make a data analysis, and perfusion units (PU) were used to assess the intensity of blood flow. The scan was performed for three times and the mean of each animal was used for statistical analysis.

### Wound Tensile Strength Measurement

The tensile strength (TS) testing device is based on a specially shaped horizontal arm pulling one side of a sample with the opposite side fixed to a measuring tip of a force meter unit (OMEGA Engineering, Inc., Stamford, CT, United States). The moving arm is driven by a high-precision stepper motor MDI-17 (Intelligent Motion Systems, Inc., Marlborough, CT, United States) through a linear slider.

The measurement technique was described previously (Gál et al., 2009). Briefly, two 1-cm-wide skin strips were removed from each incision and placed lengthwise between the clamps of the TS testing device. Pulling was performed perpendicularly to the original direction of the wound. The maximal breaking strength was recorded for each sample. The TS was then calculated by using the formula: TS = MRS/A (MBS = maximal rupture strength [g], A = wound area [mm^2^]) and expressed in g/mm^2^.

### Histological Analysis

In each group, six tissue samples were collected to perform Hematoxylin and eosin (H&E) staining. These specimens first received fixation in 4% (v/v) paraformaldehyde overnight and then were embedded in paraffin wax. After that, the specimens were cut into 5 µm thick slices for H&E staining, and then observed under an optical microscope to assess histological changes. From each section, the microvessels were measured on six fields that were selected randomly, and the number of microvessels per unit area (/mm^2^) was counted to quantify the level of microcirculation.

### Immunofluorescence

For the purpose of cell immunofluorescence, HUVECs were grown on a 6-well plate for 24 h, exposed to 0, 25 or 50 μM TF for 24 h, and then exposed to 500 μM TBHP for 2 h. After that, they were rinsed with PBS, fixed with 4% paraformaldehyde for 15 min, permeabilized with 0.1% TritonX-100 in PBS for 10 min, and blocked with 5% bovine serum albumin (BSA) for 1 h at RT. After that, they were exposed to primary antibodies against Nrf2 (1:200; Abcam) overnight at 4°C, followed by exposure to Alexa Fluor^®^488-goat or Alexa Fluor^®^594-goat anti-rabbit IgG (H + L) secondary antibodies (1:300; Jackson Immunoresearch, PA, United States) at 37°C for 1 h. Subsequently, they were stained with DAPI (Beyotime, China) for 5 min, and observed under a Nikon ECLIPSE Ti microscope (Nikon, Japan). For each slide, six arbitrary fields were analyzed. Later on, fluorescence quantification was done with Image-Pro Plus (Media Cybernetics, Rockville, MD, United States) and then analyzed by researchers who were ignorant of the experimental information.

For tissue immunofluorescence, the methods were similar to those mentioned in the immunohistochemistry procedure. As mentioned above, the tissue samples were deparaffinized, rehydrated, and washed with PBS. Endogenous peroxidase was quenched by using 3% hydrogen peroxide and the tissue antigen was repaired with 10.2 mM sodium citrate buffer. The specimens were exposed to primary antibody against a-SMA (anti-alpha smooth muscle actin (αSMA), 1:200) overnight at 4°C. After that, tissue sections were treated with a Texas red-conjugated anti-IgG secondary antibody. The nuclei were stained with DAPI. Observed under a fluorescence microscope (Olympus Inc., Tokyo, Japan).

### Immunohistochemistry

For immunohistochemistry analyses, the dewaxed and hydrated sections were placed in boiling buffer (citrate buffer; pH 6.0) for 10 min. Each section was then incubated in one drop of 3% H_2_O_2_ for 10 min and in one drop of primary monoclonal antibodies of CD31 (1:100) or VEGF (1:100) at 4°C overnight. The sections were washed with PBS, incubated with HRP goat anti-rabbit (1:400; Abcam) for 1 h, washed again with PBS and incubated with a drop of a DAB chromogen kit (ZSGB-BIO, Beijing, China), then counterstained with hematoxylin (Beyotime Institute of Biotechnology, China) for 5 min. Sections were then counterstained, differentiated and blued. Finally, the sections were dehydrated, dried, sealed, aired and photographed using a Nikon ECLPSE 80i (Nikon, Japan), and analyzed by Image-Pro Plus 6.0 software.

### Molecular Modeling

**FIGURE 1:**
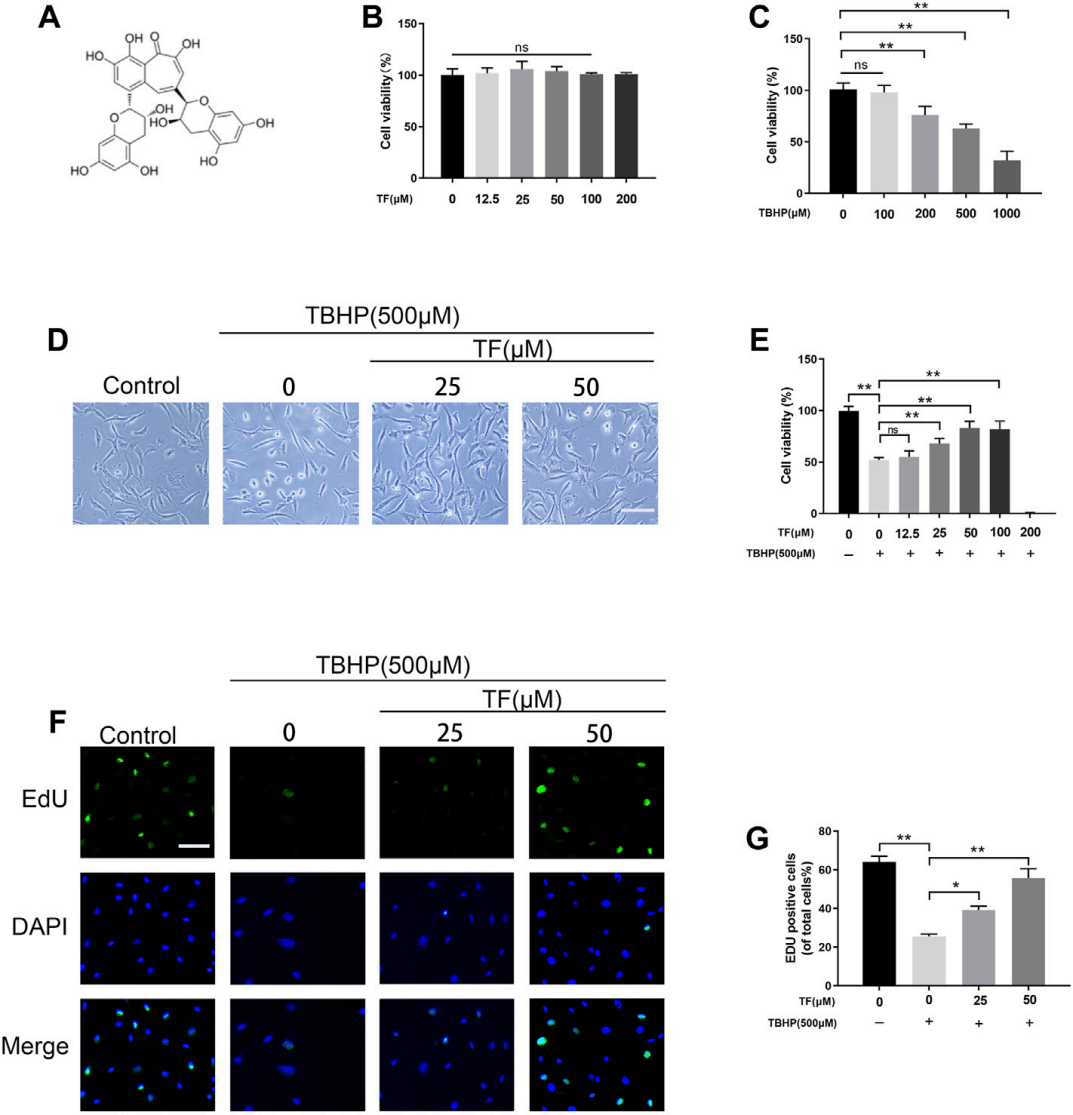
Effects of TF on cell viability and proliferation. **(A)** TF chemical structure. **(B)** Different concentrations of TF treated with HUVECs for 24 h using CCK-8 assay to assess viability. **(C)** HUVECs were exposed to different concentrations of TBHP for 24 h using CCK-8 assay to examine cellular survivability. **(D)** Representative images demonstrating cell morphology alterations in TF (0, 25, 50 μM) with or without TBHP (500 μM) co-treatment in HUVECs (scan bar, 100 μm). **(E)** Evaluation of HUVECs viability, using CCK-8 assay, after TF (0, 25, 50 μM) with or without TBHP (500 μM) co-treated in HUVECs. **(F)** Representative images show that EdU staining assay results in TF (0, 25, 50 μM) with or without TBHP (500 μM) co-treatment in HUVECs. Scale bar: 50 μm. **(G)** Histogram plots show that percentage of EdU positive cells in each group. Data presented as mean ± SD, *p <0.05, **p < 0.01, versus the indicated group, *n* = 3.

We next assessed whether there was any affinity between TF and Nrf2 or upstream proteins in the Nrf2 pathway via a computational molecular docking analysis (Luo et al., 2020). For this analysis, we utilized the TF chemical structure shown in Figure 1A. After examination of all generated models, we found TF to clearly interact with and dock in the Nrf2 binding site (Figure 4A), with macro- and local-level views of these interactions shown using a ribbon model. We additionally utilized a space-filling model to illustrate between TF and the Asp422, Val420, Val608, and Val561 residues of Nrf2. These results suggested that TF may function to inhibit the development of wound healing in part through its ability to interact with Nrf2 in a manner that promotes its nuclear translocation.

### Statistical Analysis

Data are represented by means ± standard deviation. Statistical analyses were conducted via GraphPad Prism (United States) (one-way analysis of variance (ANOVA) and Tukey’s post hoc test. Comparisons of mean values between two groups were performed using the independent-sample *t* test. *p* values < 0.05 was regarded as significant difference.

## Results

### Effects of TF on Cell Viability and Proliferation

To estimate whether TF exerts cytotoxic effects on HUVECs, we treated HUVECs with different doses of TF. No damaged cells were found in HUVECs when the dose of TF was below 50 μM. According to our data, 50 μM TF was selected as the maximum non-toxic concentration for subsequent experiments (*p* = 0.03, Figure 1B). Then, after TBHP was administered, the cell viability decreased, and 63.0 ± 4.3% cell viability was observed at the concentration of 500 µM (*p* = 0.008, Figure 1C). In subsequent experiments, compared with the TBHP-treated group, TF protected the cells from TBHP-induced damage in a dose-dependent manner (61.6 ± 2.8%, 70.0 ± 5.7%, 84.3 ± 5.2%, respectively, at the doses of 12.5, 25, and 50 µM, indicating that TF produced a cytoprotective effect on TBHP-mediated cytotoxicity (Figures 1D,E). Futhermore, 5-ethynyl-2′-deoxyuridine (EdU) fluorescence assays was used to detect TF induces proliferation in HUVECs under different conditions. The results indicated that HUVECs proliferation was dramatically reduced by TBHP but reversed by TF treatment in a dose-dependent manner (62.0 ± 3.8%, 22.6 ± 4.8%, 37.2 ± 3.8%, 58.8 ± 5.2%,respectively) (Figures 1F,G).

### TF Ameliorated Mitochondrial Functional Damage and Apoptosis in TBHP-Induced HUVECs

**FIGURE 2:**
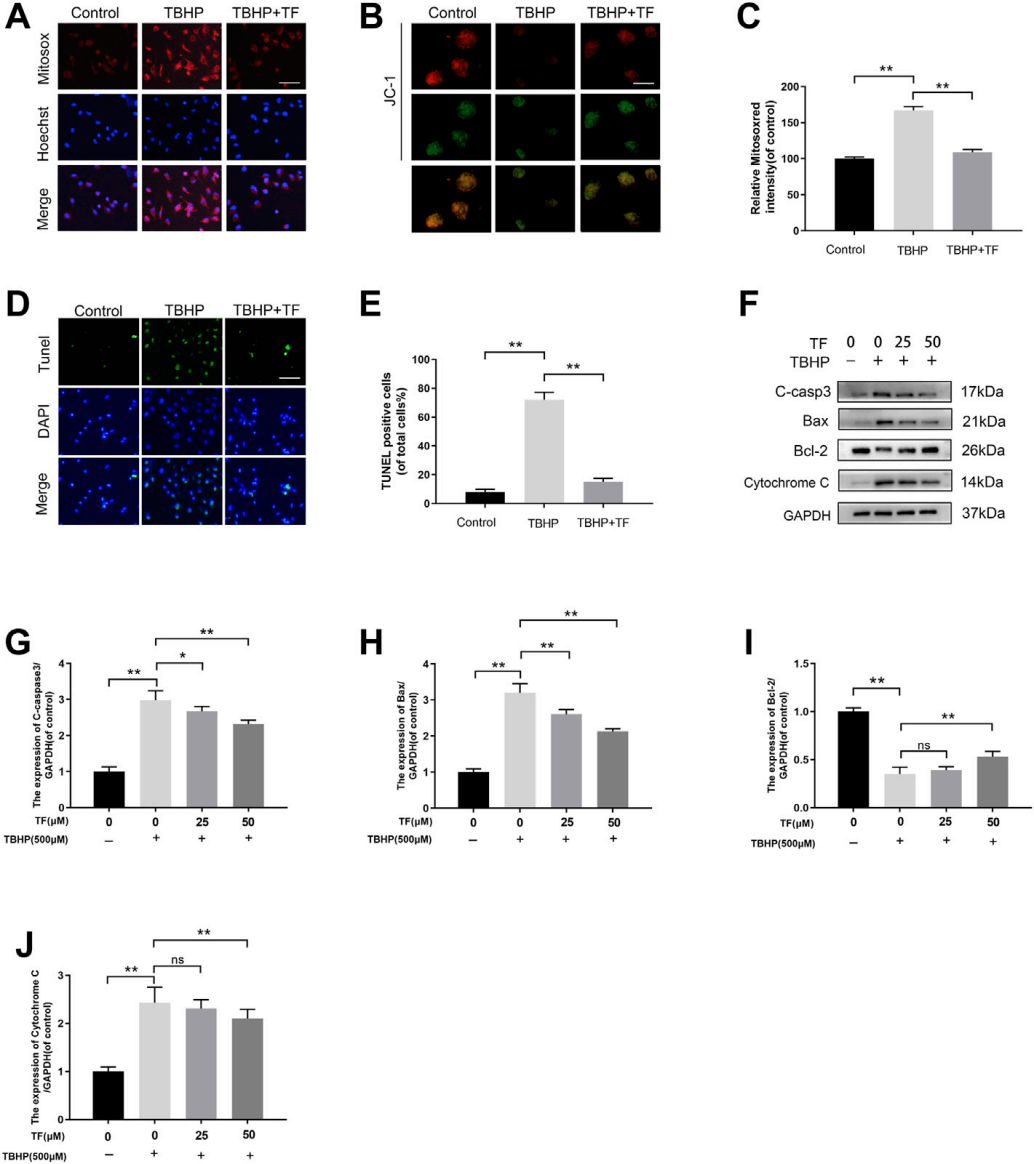
TF Ameliorated Mitochondrial Functional Damage and Apoptosis in TBHP-induced HUVECs. **(A)** HUVECs (1 × 10^4^ cells/mL) treated with or without TF (50 μM) for 24 h and TBHP (500 μM) for 2 h, then staining with 2 µM MitoSOX Red for 10 min (Bar: 20 μm). **(B)** The mitochondrial membrane potential of cells was measured via JC-1 staining. (JC-1 exists in the form of polymers in the mitochondria of cells, showing bright red fluorescence, after the mitochondrial membrane potential is reduced by TBHP treatment, JC-1 cannot exist in the form of polymers in the mitochondrial matrix. At this time, the red fluorescence intensity in mitochondria was significantly reduced, while the green fluorescence in cytoplasm was significantly enhanced) (Bar: 20 μm). **(C)** Percentage of Mitosox Red staining intensity in each group. **(D)** TUNEL assay was used to examine DNA damage in HUVECs treated with or without TF (50 μM) for 24 h and TBHP (500 μM) for 2 h. Representative images demonstrating terminal deoxynucleotidyl transferase deoxyuridine triphosphate nick end labeling-positive nuclei (green color) (scan bar, 100 μm). **(E)** Percentage of TUNEL^+^ cells in dermal layer. **(F)** The immunoblotting of cleaved-caspase3, Bax, Bcl-2, and Cytochrome C levels in HUVECs treated with TF (0, 25, 50 μM) for 24 h and presence/absence TBHP (500 μM) for 2 h. **(G–J)** Quantification of cleaved-caspase3, Bax, Bcl-2, and Cytochrome C expressions in each group. Data presented by mean ± SD, Significance: *p <0.05, **p < 0.01, versus the indicated group, *n* = 3.

Oxidative stress played a significant role in wound healing during the ischemia-reperfusion process. The high levels of oxidative stress and mitochondrial dysfunction can severely influence the functional state of HUVECs. For this reason, we evaluated the effects of TF in TBHP-treated huvec on mitochondrial dysfunction and antioxidant enzyme activity. The levels of mitochondrial ROS production were tested by MitoSox staining. According to Mitosox Red staining, compared with the untreated group, the mtROS of TBHP treatment group was 168.0 ± 7.1% (*p* = 0.0012). We also discovered that the TBHP-stimulated cells treated with TF had a lower MMP than that of the TBHP treatment group (*p* = 0.0021). Compared with the untreated cells, TBHP treatment facilitated the production of superoxide anion. These effects were restored to near physiological levels by TF treatment (Figures 2A,B). Futhermore, TUNEL assay was used to evaluate the TF-induced cytoprotection of TBHP-induced HUVECs. The expression of TUNEL^+^ cells was upregulated after TBHP treatment and alleviated depending on the dose of TF (9.9 ± 3.2%, 75.3 ± 5.9%, 22.0 ± 4.8%, respectively, *p* = 0.003, *p* = 0.002, Figures 2D,E). According to the western blot data, the results of the expressions of apoptosis-related proteins showed that TBHP-induced mitochondria dysfunction in cells was restored by TF treatment in a dose-dependent manner (Figures 2F–J).

### TF Promoted Cell Function in TBHP-Treated HUVECs

**FIGURE 3:**
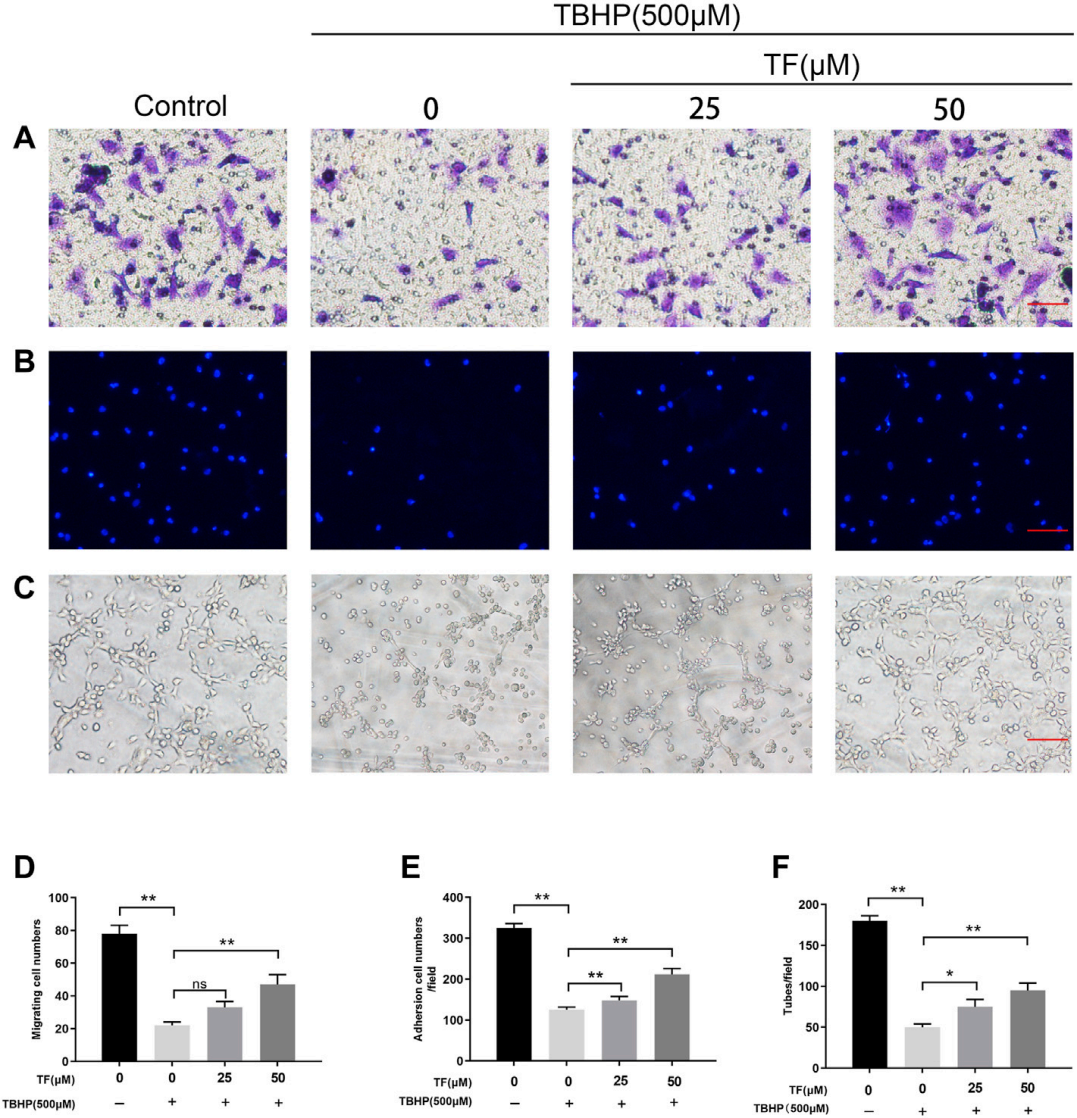
TF Restored Cell Function in TBHP-Treated HUVECs. HUVECs were treated with TF (0, 25, 50 μM) for 24 h and presence/absence TBHP (500 μM) for 2h, then treated HUVECs were performed the following series of experiments. **(A,D)** Assessment of TF-mediated HUVECs migration using transwell system. Scale bar, 100 μm. **(B,E)** Evaluation of TF-mediated HUVECs adhesion using cell-matrix adhesion assay. Scale bar, 100 μm. **(C,F)** Evaluation of TF-mediated HUVECs neovascularization using tube formation assay. Scale bar, 100 μm. Data presented as mean ± SD, *p <0.05 and **p < 0.01, versus the indicated group, *n* = 3.

To determine whether TF had therapeutic benefits on cell functions, we did several cell function experiments on the TBHP-treated HUVECs. Firstly, a transwell migration assay was performed to examine potential huvec migration mediated by TF. As shown in Figure 3, there were typical representations of hematoxylin-stained cells that migrated to the basolateral membrane. The statistical results showed that TBHP treatment significantly lowered the number of migratory cells, while TF reversed this effect (*p* = 0.001, *p* = 0.003, *p* = 0.006, respectively, Figures 3A,C). Next, to evaluate the role of TF on huvec adhesion, a fibronectin adhesion assay was conducted. The statistical analysis indicated that TBHP stimulation significantly reduced the number of adherent cells, but this effect was reversed by TF treatment (*p* = 0.002, *p* = 0.005, *p* = 0.007, respectively, Figures 3B,D). Lastly, a tube formation assay was used to examine huvec neovascularization after TF treatment, and then the number of capillary-like structures within each group was observed. Based on our data, TBHP markedly reduced huvec neovascularization, while TF pretreatment can protect from the TBHP treatment in a dose-proportional manner (*p* = 0.012, *p* = 0.004, *p* = 0.006, respectively, Figures 3C,F).

### TF-Mediated Cytoprotection via Promoting Nucleus Translocation of Nrf2 and Activating the PI3K/AKT/Nrf2 Signaling Pathway

**FIGURE 4:**
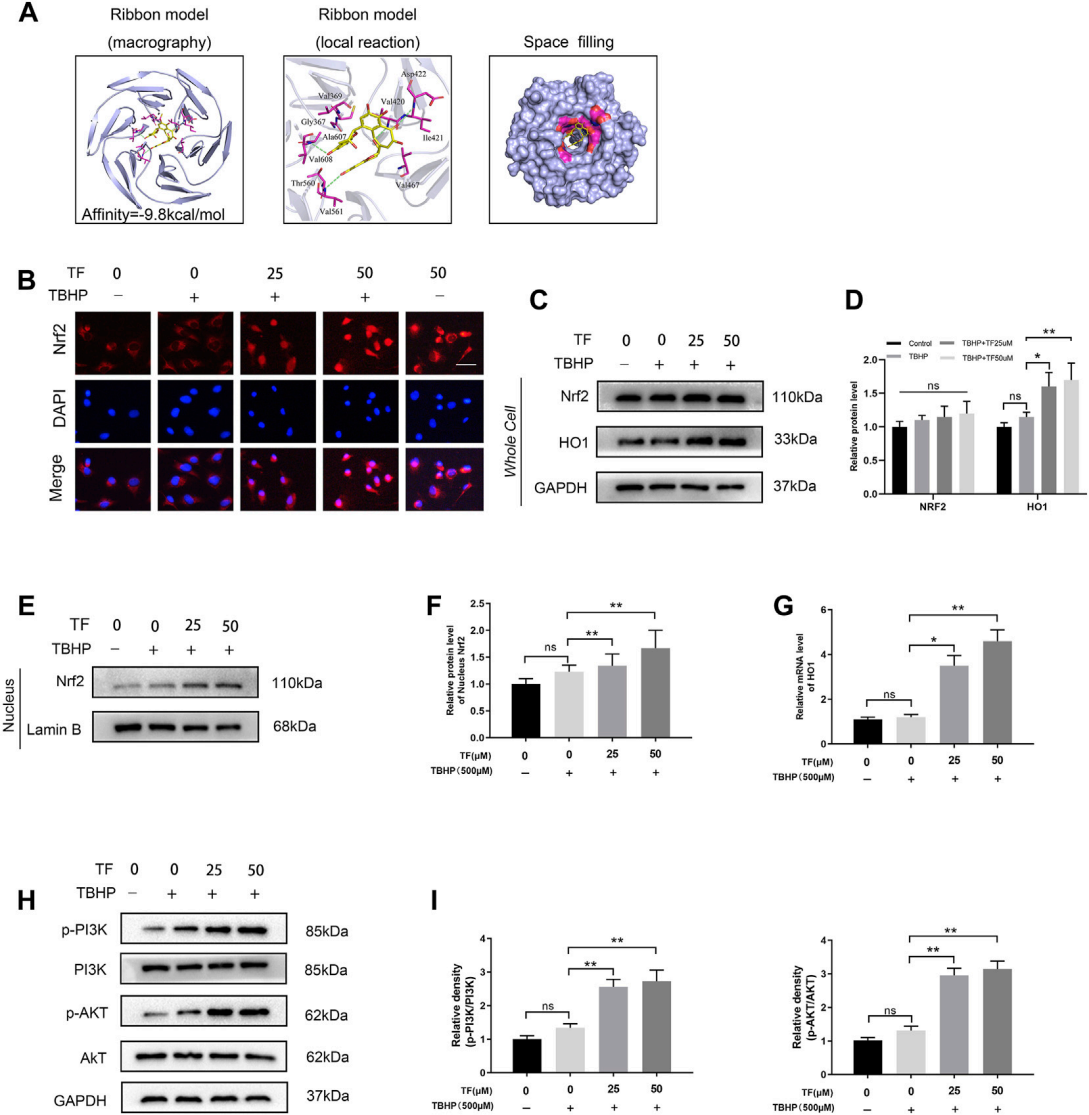
TF-mediated Cytoprotection via promoting nucleus translocation of Nrf2 and activating the PI3K/AKT/Nrf2 signaling pathway. **(A)** A ribbon model is applied for representing protein residues and illustrates a 3D binding model. Binding site affinity = -9.8 kcal/mol exists in TF docking with Nrf2. In this predicted model, TF interacts with Asp422、Val420、Val608 and Val561 on Nrf2. A space filling model was used to show the TF binding in the Nrf2 pocket. **(B)** Representative immunofluorescence images of HUVECs treated with TF (0, 25μM, 50 μM) for 24 h and with/without 500 μM TBHP for 2 h showing Nrf2 expression in the HUVECs. Scale bar: 20 μm. **(C,D)** Expression of Nrf2 and HO-1 levels in the cells and analysis of the optical density values as depicted above. **(E,F)** Expression of Nrf2 in the nuclei of HUVECs treated with TF (0, 25, 50 μM) for 24 h and presence/absence TBHP (500 μM) for 2 h performed by western boltting and analysis of the optical density values of Nrf2 in cells treated as depicted above. **(G)** QRT-PCR analysis shows the HO1 mRNA levels in HUVECs treated with TF (0, 25, 50 μM) for 24 h and presence/absence TBHP (500 μM) for 2 h. **(H,I)** Expression of p-PI3K, PI3K, p-AKT and AKT in the HUVECs treated with TF (0, 25, 50 μM) for 24 h and presence/absence TBHP (500 μM) for 2 h and analysis of the optical density values as depicted above. Data represented as mean ± SD, *p <0.05 and **p < 0.01, versus the indicated group, *n* = 3.

To determine whether TF and Nrf2 or other proteins in the Nrf2 axis exists any affinity, a artificial intelligence software was utilized to simulate molecular docking (Luo et al., 2020). We applied the TF chemical structure (Figure 1A) for further analysis. Upon establishment of all possible models, TF does exist clearly intract and dock with the Nrf2 docking site (Figure 4A). We adapted the ribbon model to display the macro- and local-level views of these interactions. Moreover, a space-filling model was performed to demonstrate this interaction. Finally, we found that there was a high-affinity (−9.8 kcal/mol) hydrogen binding events between TF and Nrf2 residues, incluing Asp422, Val420, Val608, and Val561. Results of software simulation indicated that TF may influence the wound healing process through its ability to interact with Nrf2, thereby promoting its nuclear translocation. Since Nrf2 is an important transcription factor in the redox reaction system (Kanlaya et al., 2021), we explored the potential role of Nrf2 played in TF-induced the cellular protective effect in HUVECs exposed to TBHP-induced oxidative stress. In order to determine whether TF promoted nuclear translocation of Nrf2 in the HUVECs, we carried out immunofluorescence staining on Nrf2, which suggested that TF induced the nuclear translocation of Nrf2 in the HUVECs (Figure 4B). Furthermore, the western blotting further confirmed that compared with the control, there were higher levels of Nrf2 in the nuclei of TF-treated HUVECs (*p* = 0.037, *p* = 0.004, respectively, Figures 4E,F). Later, we analyzed the expression level of HO1, a target protein of Nrf2. According to the expression of HO1 mRNA and western blotting, compared with the control group, TF-treated HUVECs significantly facilitated the expression of HO1 (Figures 4C,D,G). In the end, we examined whether TF resulted in different expressions of Nrf2 upstream molecules, including Akt and PI3K. In keeping with previous results, TF significantly promoted phosphorylation of Akt and PI3K (Figures 4H,I). These results implied that TF increased the nuclear transcription of Nrf2 in HUVECs by activating AkT and PI3K.

### The Cytoprotective Effect of TF is Eliminated by the Nrf2 Knockdown

**FIGURE 5:**
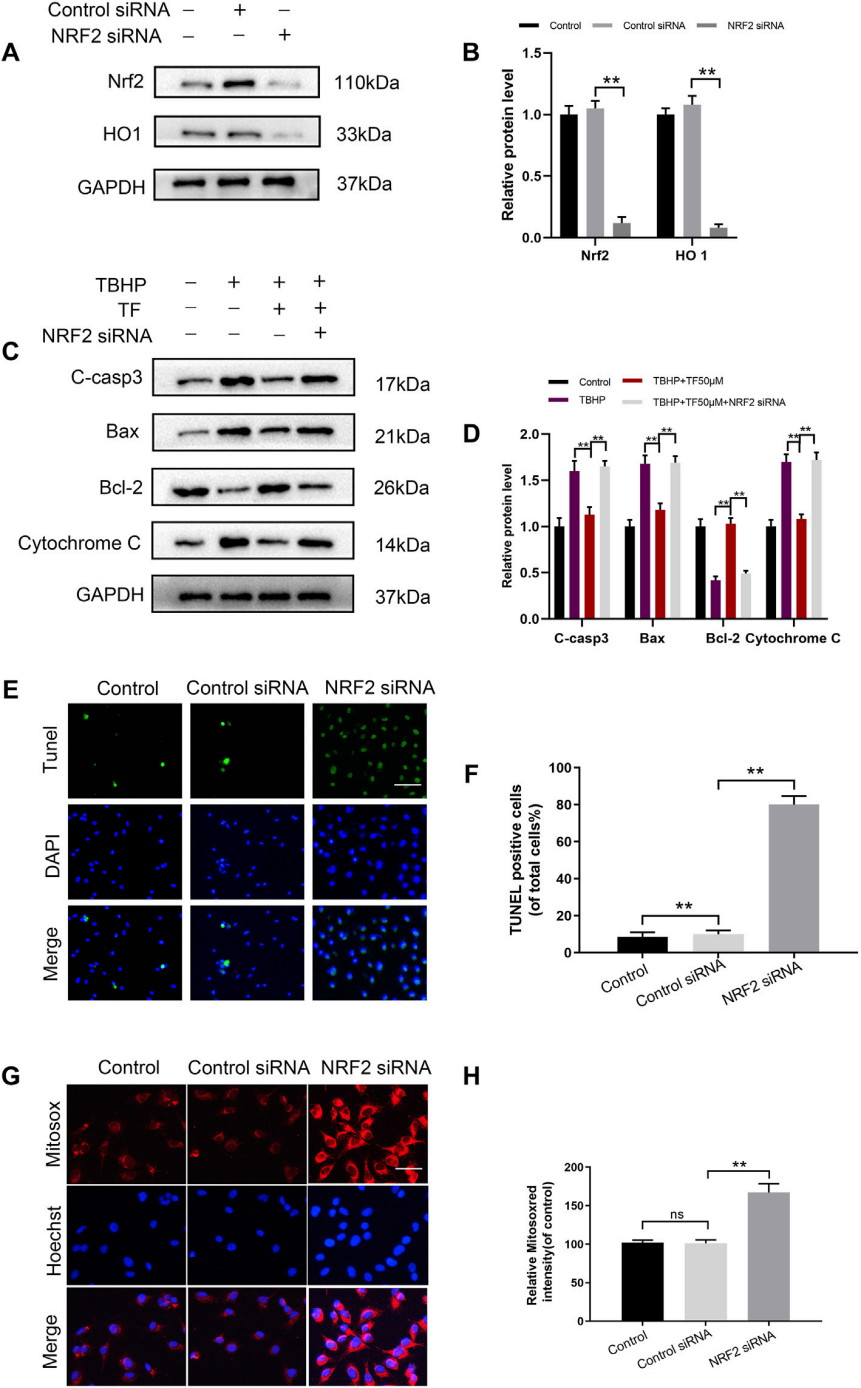
Nrf2 knockdown abrogates the beneficial effects of TF. **(A,B)** Western blot analysis shows Nrf2 and HO1 protein levels in control and Nrf2 knockdown HUVECs. **(C)** Representative western blot images and **(D)** Histogram plots show the levels of C-casp3, Bax, Bcl-2 and Cytochrome C proteins in the control and Nrf2 knockdown HUVECs treated with or without TF (50 μM) for 24h and 500 μM TBHP for 2 h. **(E,F)** Percentage of TUNEL^+^ cells in the dermal layer, after Nrf2 siRNA incorporation and treatment as depicted above (scan bar, 100 μm) (nuclei, blue; positive cells, green). **(G,H)** The cells (10^4^/ml) were stained with 2 µM MitoSOX Red for 10 min and fluorescence intensity was measured using a fluorometer in control and Nrf2 knockdown HUVECs. Percentage values were calculated compared to those in the untreated group (Bar: 20 μm). Data represented as mean ± SD, *p <0.05 and **p < 0.01, versus the indicated group, *n* = 3.

To explore whether TF induced cytoprotection by activating PI3K/AKT/Nrf2 signaling pathway, we silenced Nrf2 by using siRNAs and determined its effect in TF-treated HUVECs. As evidenced by the results of western blotting, the levels of Nrf2 and HO1 proteins in the control group were higher than those in si-Nrf2-transfected HUVECs (Figures 5A,B). Furthermore, as expected, Nrf2 siRNA reversed TF-induced alteration in apoptosis-related proteins. Compared with the control group, the western expression levels of C-caspase3, Bax, Bcl-2 and Cytochrome C significantly decreased in the Nrf2-silenced TF-induced HUVECs (Figures 5C,D). The TUNEL assay also confirmed that Nrf2 siRNA abrogated the TF-mediated protection of HUVECs (Figures 5E,F). Futhermore, MitoSox Red staining were significantly increased in the Nrf2-silenced TF-treated HUVECs compared to controls (Figures 5G,H). Taken together, these results showed that the protective effect of TF can be eliminated by Nrf2 knockdown.

### TF Promotes Wound Healing Process in Rats

**FIGURE 6:**
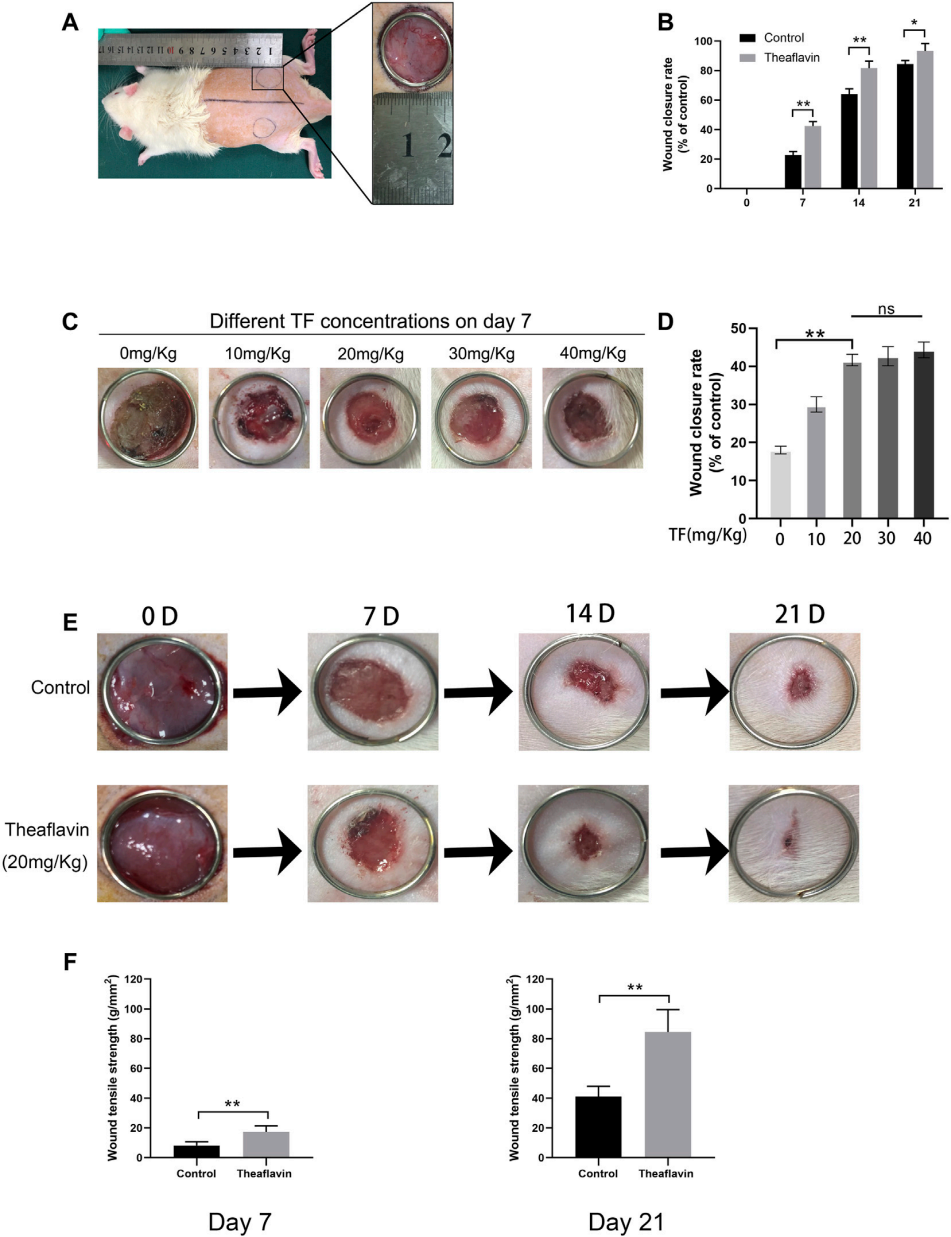
TF Promotes wound healing process in rats. **(A)** Two round dermal wounds were established bilaterally on rat dorsal trunk as mentioned above. **(B)** Wound closure rates in the control and TF (20 mg/kg) group at different times. Healing rates of full-thickness cutaneous wounds were significantly increased by the TF treatment. **(C)** Effects of different doses of TF on POD 7. **(D)** Wound closure rates in the different doses of TF. **(E)** Representative digital images of wound closure for control and TF (20 mg/kg) group on day 0, day 7, day 14, and day 21. Data represented as mean ± SD, *p <0.05 and **p < 0.01, n = 5. **(F)** Wound tensile strengths of wounds removed from untreated control, TF-treated (20 mg/kg) groups at day 7 and 21 post-surgery. The data are presented as the means ± SD of five independent experiments. *p < 0.05, **p < 0.01, versus the indicated group, *n* = 5.

According to our *in vitro* experiments, TF played a role in promoting angiogenesis. We hypothesized that it can also contribute to angiogenesis and accelerate wound healing process. To this end, we established a rat model of full-thickness skin wound to assess the effect of TF on wound repair after surgery. The surgical process of wounding is shown in Figure 6A. First, thirty rats were administered different doses of TF to determine the appropriate dose for wound healing (Figure 6C). As seen in Figure 6D, the wound closure rate increased with the increase of TF doses from 0 to 20 mg/kg, whereas it was no significance with administered dose of TF from 20 to 40 mg/kg. So 20 mg/kg was determined as the appropriate dose for wound healing in subsequent experiments. On day 7, the closure rate of wounds was 42.5 ± 2.3% in the TF treatment group and 21.3 ± 1.7% in the control group respectively. On day 14, the healing in the TF treatment group accelerated and the closure rate remained significantly higher than the control group. On day 21, the wounds in the TF treatment group were nearly closed, while part of the wounds in the control group remained unhealed (*p* = 0.001, *p* = 0.003, *p* = 0.042, respectively, Figures 6B,E), indicating that TF accelerated wound healing *in vivo*. The results of wound tensile strength measurements on day7 and day21 are shown in Figure 6F. On day 7 after wounding, the tensile strength (TS) of wound significantly increased in the TF group (TF = 17.2 ± 3.3 g/mm^2^), but only 5.9 ± 2.5 g/mm^2^ in the control group. On the other hand, on day21, compared with the control, the TS of TF-treated skin wounds was significantly higher (TF = 83.1 ± 12.2 g/mm^2^, control = 42.3 ± 8.4 g/mm^2^). Taken together, there was a significant improvement in TS after 3 weeks in the TF treatment group than in the untreated group.

### TF Enhanced Angiogenesis in Wound Healing Process

**FIGURE 7:**
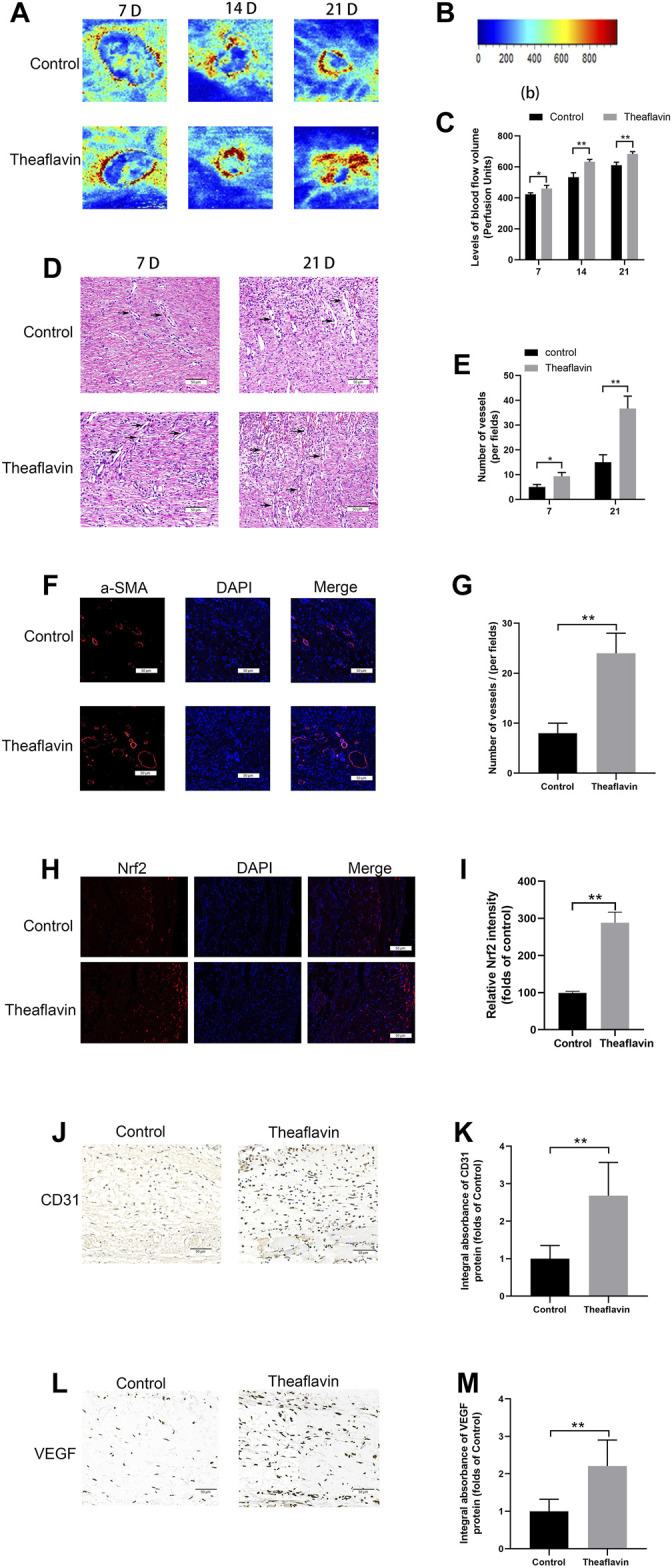
TF enhanced angiogenesis in wound healing process. (**A)** Evaluation of Blood flow and vascular distribution by LDBF in the control and TF (20 mg/kg) group. **(B)** Relative intensity bar of blood flow signal. **(C)** Quantification of signal intensities of blood flow volume at day 7, day 21. **(D)** The H&E staining showing the control and TF (20 mg/kg) group subcutaneous blood vessels (black arrow) and inflammation at day 7, 21 (original magnification ×200; scan bar, 50 μm). **(E)** Histogram depicts mean vessel density in the flap (/mm^2^). **(F)** Immunofluorescence staining of a-SMA in vascular endothelial cells in the control and TF (20 mg/kg) group (original magnification ×200; scan bar, 50 μm). **(G)** Histogram shows that a-SMA positive vessel density in dermal layer. **(H)** Immunofluorescence staining of Nrf2 in the control and TF (20 mg/kg) group (original magnification ×200; scan bar, 50 μm). **(I)** Statistical chart of the percentage of the Nrf2-positive cells. **(J)** Representative images of CD31 immunohistochemical staining at Day 7 post operation (scale bar, 50 μm). **(K)** Quantification of integral absorbance of CD31 in IHC. **(L)** Representative images of VEGF immunohistochemical staining at Day 7 post operation (scale bar, 50 μm). **(M)** Quantification of integral absorbance of VEGF in IHC. Data represented as mean ± SD, *p <0.05 and **p < 0.01, versus the indicated group, *n* = 5.

To detect whether TF exerted a prominent effect on the microvascular network on the back of rats, we performed Laser Doppler to visualize the circulatory system. On day 7, the results suggested that compared with the control group, TF group had a markedly stronger signal intensity in the flaps (422.1 ± 8.64PU, 460.6 ± 16.3PU, respectively, *p* = 0.004, Figures 7A,C). On day 14, the control group was 533.3 ± 23.57PU and the TF group was 633.3 ± 12.47PU. Eventually, on day 21, the control group was 610.2 ± 16.32PU and the TF group was 683.3 ± 12.47PU. In addition, we carried out hematoxylin-eosin staining on day 7 and day 21, and the results indicated that compared with control rats, the vessel density in the TF rats was significantly improved (*p* = 0.0014, *p* = 0.003, respectively, Figure 7E). Compared with control rats, the immunofluorescenc staining of α-SMA in the TF rats also significantly enhanced, which coincided with the results of H&E staining (*p* = 0.005, Figure 7G). In immunofluorescence, a large number of Nrf2 positive cells were identified in the dermis of the TF group relative to the control group, as depicted in Figures 7H,I (*p* = 0.002). IHC was employed to detect the expression of CD31. Compared with the control group, the CD31 expression was considerably enhanced in the TF group, as shown in Figures 7J,K. Similarly, the quantity of VEGF-positive vessels in TF-treated animals were obviously higher than that of the controls (Figures 7L,M). Collectively, these data showed that TF faciliated angiogenesis and played a substantial role in improving wound healing.

### Different Ligands Docked in the Nrf2 Binding Site

We also tested the affinity of other molecules known to bind Nrf2, as shown in the Table 1. This affinity is an important indicator to measure whether the ligand can effectively bind to the receptor molecule, and it is the core parameter of AutoDock Vina software. The lower the affinity value is, the closer the ligand binds to the receptor. From the Table 1, we found that the affinity of these known molecules was close to TF, indicating that TF had a high affinity with Nrf2.

**TABLE 1:** Different ligands docked in the Nrf2 binding site. The lower the affinity value is, the closer the ligand binds to the receptor.

Protein	Grid_size	Ligand	Docking score (Kcal/mol)
NRF2	40 × 40 × 40	Limomin	−9.6
		Proanthocyanidins	−9.4
		Paeoniflorin	−9.2
		Theaflavin	−9.8

## Discussion

In our study, we have firstly certified that Theaflavin (TF) exposure stimulated the antioxidant PI3K/AKT/Nrf2 signaling axis followed by attenuating cellular apoptosis and ameliorating the oxidative-induced celluar dysfunction on HUVECs. In our *in vivo* experiment, the gastric delivery of TF (20 mg kg^−1^·day^−1^) significantly accelerated the wound healing process, which was characterized by faster wound closure, increased new capillaries. As TF could enhance the formation of new capillaries at the wound site and promote wound healing *in vivo*, it can be considered as a promising drug for the treatment of ischemic wounds.

The healing process of wounds involves a series of intricate events. And the healing process of wound is mediated by plenty of individual growth factors, cytokines, and hormones (Barrientos et al., 2008; Behm et al., 2012; Ridiandries et al., 2018). In our study, the ability for HUVECs proliferation, adhesion, migration and tube formation was regulated by TF treatment, demonstrating that TF had proangiogenic effects. In general, the angiogenic process could be divided into a few stages, including endothelial cell proliferation, separation and migration, adherence to the ECM and differentiation (Lamalice et al., 2007). In our present study, TF could evidently boost HUVECs proliferation, which can be regarded as a signal of the beginning of angiogenesis. In addition, TF could regulate the adhesion of HUVECs, with decreased cell–cell adhesion and increased cell-matrix adhesion. Finally, endothelial cells differentiate into new capillary vessels, which are confirmed by the faster tube formation rate and tube numbers (Figures 3C,F). Taken together, TF exerted effective in pro-angiogenesis *in vitro*.

In our study, we discovered that TF (0–50 μM) didn’t produce a detrimental effect on cell viability. And TBHP was applied to induce an oxidative stress state in HUVECs. In conformity to other publications, we observed that at a concentration of 500 μM TBHP treatment significantly reduced the viability of HUVECs, but this effect was restored by TF pretreatment in a dose-dependent manner. This implied that when TBHP was administered, TF produced a cytoprotective effect in HUVECs. What’s more, we also found that TF pretreatment can downregulate the levels of apoptosis-related protein Bax, Cytochrome C and C-caspase3, and upregulate the level of the anti-apoptotic protein Bcl-2. In normal physiological environments, mitochondrial membrane potential equilibrium is strictly controlled. However, TBHP induces apoptosis by increasing the production of mtROS associated with the interruption of mitochondrial membrane potential, which facilitates the release of cytochrome C from mitochondria to cytoplasm by activating apoptotic protein Bax and inactivating the anti-apoptotic protein Bcl-2 (Ju et al., 2017). The results of this study suggested that TF protected HUVECs from TBHP-induced apoptosis by inhibiting the production of mtROS and inhibiting caspase, implying that TF was an effective antioxidant that can protect HUVECs from oxidative stress. Our results also demonstrated that TF-pretreatment was able to significantly improve cellular dysfunction induced by TBHP stimulation on HUVECs.

We also tested the affinity of other molecules known to bind Nrf2, as shown in the Table1. This affinity is an important indicator to measure whether the ligand can effectively bind to the receptor molecule, and it is the core parameter of AutoDock Vina software. The lower the affinity value is, the closer the ligand binds to the receptor. From the Table1, we found that the affinity of these known molecules was close to TF, indicating that TF had a high affinity with Nrf2.

HO1 offers cellular protection in the treatment of vascular disease (Durante, 2010). HO1 is ubiquitous in the heart and blood vessels, protects from vasculopathy and offers cytoprotection for endothelial cells (Khitan et al., 2014; Sandrim et al., 2019). Mice, whose HO-1 gene is knocked down, are very vulnerable to severe vascular diseases (Hull et al., 2013). Nrf2 is widely accepted as an essential transcriptional agent, and its stimulation can increase the levels of downstream antioxidant-related genes, including HO1(Park et al., 2017; Huang et al., 2018; Liu et al., 2018). In a normal internal environment, Kelch-like ECH-associated protein-1 (Keap1) marks Nrf2 for ubiquitin-mediated proteasomal destruction (Suzuki and Yamamoto, 2015; Bellezza et al., 2018). In the presence of an external stimulus, Keap1 becomes inactivated and the ubiquitination-mediated removal of Nrf2 is weakened, leading to the upregulation of nuclear Nrf2 and combination with antioxidant response factors (Nezu et al., 2017; Guo and Mo, 2020). This process enhances the levels of antioxidants and second-stage detoxification enzymes like HO1. Oxidative stress can stimulate the Nrf2/HO1 axis, a key determinant of homeostasis maintenance in case of oxidative stress damage (Lv et al., 2019; Wu et al., 2019). However, after co-administration with TF, the HO1 levels and nuclear accumulation of Nrf2 were upregulated in TBHP-induced HUVECs, while the apoptosis and celluar dysfunction persisted. This was probably because TBHP treatment stimulated of the Nrf2/HO1 axis improperly, so as to circumvent cytotoxicity. Multiple studies have suggested that in specific cell types, TF alleviated the oxidative stress-driven injury through the Nrf2/HO1 axis (Cheng et al., 2013; Li et al., 2021). In fact, our data revealed that TF significantly upregulated the nuclear accumulation of Nrf2 and HO1 in TBHP-stimulated HUVECs. Nrf2 siRNA merger was performed to inhibit the expression of Nrf2, then decrease the levels of HO-1, and increase the levels of apoptosis-associated proteins. Based on these data, TF-mediated cytoprotection of TBHP-treated HUVECs took advantage of the stimulation of Nrf2/HO-1 axis.

PI3K/Akt is a classical signaling pathway that plays an important role in cell proliferation, migration, apoptosis, angiogenesis, and other biological processes. AKT is a serine/threonine kinase and a downstream signal molecule of PI3K, activated by it (Li et al., 2017). Several studies have reported that the PI3K/Akt pathway is essential for regulating Nrf2/Ho-1 pathway activation and is thus involved in protection against oxidative stress and apoptosis in multiple cell types (Di Tu et al., 2020; Feng et al., 2021; Wu et al., 2021). Under oxidative stress, the PI3K/Akt signaling pathway leads to Nrf2-dependent transcription and overexpression of HO-1 protein (He et al., 2019). We found that phosphorylation of AKT protein increased in the presence of TF, which possibly upregulated TF-induced Nrf2/HO-1 protein. Under normal physiological conditions, Keap1 sequesters Nrf2 in the cytoplasm. After electrophilic agents or ROS oxidize cysteine residues within Keap1, Nrf2 translocates to the nucleus following dissociation from its cytoplasmic docking protein. In the nucleus, Nrf2 activates the transcription of several phase II detoxifying enzymes and antioxidant enzymes including HO-1 by binding to their promoter regions (Ryu et al., 2014). Thus, we speculate whether TF also promotes the angiogenesis by activating AKT phosphorylation. As we expected, the phosphorylated expression of AKT was enhanced and the expression of Nrf2 in the nucleus was activated under the effect of TF, indicating that AKT participated in the proangiogenic process of TF. In addition, a large number of studies have shown that Nrf2 is a redox transcription factor and a major participant in antioxidant response (Li et al., 2009). PI3K can also promote neuronal survival by activating Akt phosphorylation and Nrf2 nuclear translocation as reported (de Oliveira et al., 2016). In our study, the p-PI3K and p-AKT were increased after TF treatment, confirmed by western blot results, in which demonstrating that TF could promote angiogenesis via the PI3K/AKT/Nrf2 signaling pathway.

In an effort to evaluate the effect of TF *in vivo*, we established full-thickness cutaneous wound model, to assess the healing process of wounds on day0, day7, day14 and day21 after surgery. Our results indicated that compared with the control group, the TF treatment quickened wound healing. On day 21, the wounds in the TF treatment group were completely closed, while some wounds in the control group remained unhealed. Angiogenesis was critical for wound healing. To evaluate TF’s activity on angiogenesis, IHC staining of CD31-positive vascular cells were carried out, and it was found that TF had increased the density of blood vessels in the wound tissue. Then whether TF modulated VEGF was investigated, which were found to have enhanced the angiogenesis. VEGF contributed to multiple processes of angiogenesis (particularly mitosis of vascular cells) promoting the formation and maturation of neovascularization. In brief, our work confirmed that TF probably served as an agent for wound healing.

## Conclusion

In summary, according to the results of *in vitro* and *in vivo* experiments, we found that TF is possible target for wound healing therapy. In HUVECs, TF-mediated cytoprotection will attenuate oxidative stress-driven apoptosis and ameliorate cellular function. And the stimulation of PI3K/AKT/Nrf2 signaling axis is probably of great significance for the improvement of the healing process of wounds treated with TF.

## Data Availability

The original contributions presented in the study are included in the article/Supplementary Material, further inquiries can be directed to the corresponding author.

## References

[B1] Barrientos S. Stojadinovic O. Golinko M. S. Brem H. Tomic-Canic M. (2008). PERSPECTIVE ARTICLE: Growth Factors and Cytokines in Wound Healing. Wound Repair Regen. 16 (5), 585–601. 10.1111/j.1524-475X.2008.00410.x 19128254

[B2] Behm B. Babilas P. Landthaler M. Schreml S. (2012). Cytokines, Chemokines and Growth Factors in Wound Healing. J. Eur. Acad. Dermatol. Venereol. 26 (7), 812–820. 10.1111/j.1468-3083.2011.04415.x 22211801

[B3] Bellezza I. Giambanco I. Minelli A. Donato R. (2018). Nrf2-Keap1 Signaling in Oxidative and Reductive Stress. Biochim. Biophys. Acta (Bba) - Mol. Cel Res. 1865 (5), 721–733. 10.1016/j.bbamcr.2018.02.010 29499228

[B4] Böckmann S. Hinz B. (2020). Cannabidiol Promotes Endothelial Cell Survival by Heme Oxygenase-1-Mediated Autophagy. Cells 9 (7), 1703. 10.3390/cells9071703 PMC7407143 32708634

[B5] Boer M. Duchnik E. Maleszka R. Marchlewicz M. (2016). Structural and Biophysical Characteristics of Human Skin in Maintaining Proper Epidermal Barrier Function. pdia 1 (1), 1–5. 10.5114/pdia.2015.48037 PMC4793052 26985171

[B6] Broughton G. 2nd Janis J. E. Attinger C. E. (2006). Wound Healing: an Overview. Plast. Reconstr. Surg. 117 (7 Suppl. l), 1e–S. 10.1097/01.prs.0000222562.60260.f9 16801750

[B7] Carracedo J. Buendía P. Merino A. Madueño J. A. Peralbo E. Ortiz A. (2012). Klotho Modulates the Stress Response in Human Senescent Endothelial Cells. Mech. ageing Dev. 133, 647–654. 10.1016/j.mad.2012.09.002 23000105

[B8] Cheng Y.-T. Wu C.-H. Ho C.-Y. Yen G.-C. (2013). Catechin Protects against Ketoprofen-Induced Oxidative Damage of the Gastric Mucosa by Up-Regulating Nrf2 *In Vitro* and *In Vivo* . J. Nutr. Biochem. 24 (2), 475–483. 10.1016/j.jnutbio.2012.01.010 22704780

[B9] Davidson J. D. Mustoe T. A. (2001). Oxygen in Wound Healing: More Than a Nutrient. Wound Repair Regen. 9 (3), 175–177. 10.1046/j.1524-475x.2001.00175.x 11472612

[B10] de Oliveira M. R. Peres A. Ferreira G. C. Schuck P. F. Bosco S. M. D. (2016). Carnosic Acid Affords Mitochondrial Protection in Chlorpyrifos-Treated Sh-Sy5y Cells. Neurotox Res. 30 (3), 367–379. 10.1007/s12640-016-9620-x 27083155

[B11] Di Tu Q. Jin J. Hu X. Ren Y. Zhao L. He Q. (2020). Curcumin Improves the Renal Autophagy in Rat Experimental Membranous Nephropathy via Regulating the PI3K/AKT/mTOR and Nrf2/HO-1 Signaling Pathways. Biomed. Research International 2020, 1–12. 10.1155/2020/7069052 PMC7654212 33204708

[B12] Dong H. Qiang Z. Chai D. Peng J. Xia Y. Hu R. (2020). Nrf2 Inhibits Ferroptosis and Protects against Acute Lung Injury Due to Intestinal Ischemia Reperfusion via Regulating SLC7A11 and HO-1. Aging 12 (13), 12943–12959. 10.18632/aging.103378 32601262 PMC7377827

[B13] Durante W. (2010). Targeting Heme Oxygenase-1 in Vascular Disease. Cdt 11 (12), 1504–1516. 10.2174/1389450111009011504 PMC2978667 20704550

[B14] Feng Z. Wang C. Yue J. Jin Q. Meng Q. Wu J. (2021). Kaempferol-induced GPER Upregulation Attenuates Atherosclerosis via the PI3K/AKT/Nrf2 Pathway. Pharm. Biol. 59 (1), 1106–1116. 10.1080/13880209.2021.1961823 34403325 PMC8436971

[B15] Gál P. Toporcer T. Vidinský B. Hudák R. Zcaron J. Sabo J. (2009). Simple Interrupted Percutaneous Suture versus Intradermal Running Suture for Wound Tensile Strength Measurement in Rats: a Technical Note. Eur. Surg. Res. 43 (1), 61–65. 10.1159/000219214 19451720

[B16] Gallo R. L. (2017). Human Skin Is the Largest Epithelial Surface for Interaction with Microbes. J. Invest. Dermatol. 137 (6), 1213–1214. 10.1016/j.jid.2016.11.045 28395897 PMC5814118

[B17] Gu M. Jin J. Ren C. Chen X. Pan Z. Wu Y. (2021). 20-Deoxyingenol Alleviates Osteoarthritis by Activating TFEB in Chondrocytes. Pharmacol. Res. 165, 105361. 10.1016/j.phrs.2020.105361 33460793

[B18] Guo Z. Mo Z. (2020). Keap1‐Nrf2 Signaling Pathway in Angiogenesis and Vascular Diseases. J. Tissue Eng. Regen. Med. 14 (6), 869–883. 10.1002/term.3053 32336035

[B19] Gupta M. K. Qin R. (2003). Mechanism and its Regulation of Tumor-Induced Angiogenesis. Wjg 9 (6), 1144–1155. 10.3748/wjg.v9.i6.1144 12800214 PMC4611774

[B20] Han X. Zhang J. Xue X. Zhao Y. Lu L. Cui M. (2017). Theaflavin Ameliorates Ionizing Radiation-Induced Hematopoietic Injury via the NRF2 Pathway. Free Radic. Biol. Med. 113, 59–70. 10.1016/j.freeradbiomed.2017.09.014 28939421

[B21] He S. Guo Y. Zhao J. Xu X. Song J. Wang N. (2018). Ferulic Acid Protects against Heat Stress-Induced Intestinal Epithelial Barrier Dysfunction in IEC-6 Cells via the PI3K/Akt-Mediated Nrf2/HO-1 Signaling Pathway. Int. J. Hyperthermia 35 (1), 112–121. 10.1080/02656736.2018.1483534 30010455

[B22] Huang B. He D. Chen G. Ran X. Guo W. Kan X. (2018). α-Cyperone Inhibits LPS-Induced Inflammation in BV-2 Cells through Activation of Akt/Nrf2/HO-1 and Suppression of the NF-Κb Pathway. Food Funct. 9 (5), 2735–2743. 10.1039/c8fo00057c 29667667

[B23] Hull T. D. Bolisetty S. DeAlmeida A. C. Litovsky S. H. Prabhu S. D. Agarwal A. (2013). Heme Oxygenase-1 Expression Protects the Heart from Acute Injury Caused by Inducible Cre Recombinase. Lab. Invest. 93 (8), 868–879. 10.1038/labinvest.2013.74 23732814 PMC3729748

[B24] Ilacqua A. N. Shettler J. A. Wernke K. M. Skalla J. K. McQuade K. J. (2017). Theaflavins from Black tea Affect Growth, Development, and Motility in Dictyostelium discoideum. Biochem. biophysical Res. Commun. 491 (2), 449–454. 10.1016/j.bbrc.2017.07.058 28711497

[B25] Jiang J. Dong C. Zhai L. Lou J. Jin J. Cheng S. (2021). Paeoniflorin Suppresses TBHP-Induced Oxidative Stress and Apoptosis in Human Umbilical Vein Endothelial Cells via the Nrf2/HO-1 Signaling Pathway and Improves Skin Flap Survival. Front. Pharmacol. 12, 735530. 10.3389/fphar.2021.735530 34803685 PMC8600365

[B26] Ju C. Gao J. Hou L. Wang L. Zhang F. Sun F. (2017). Neuroprotective Effect of Chondroitin Sulfate on SH-Sy5y Cells Overexpressing Wild-type or A53T Mutant α-synuclein. Mol. Med. Rep. 16 (6), 8721–8728. 10.3892/mmr.2017.7725 28990084 PMC5779948

[B27] Kanlaya R. Subkod C. Nanthawuttiphan S. Thongboonkerd V. (2021). Caffeine Prevents Oxalate-Induced Epithelial-Mesenchymal Transition of Renal Tubular Cells by its Anti-oxidative Property through Activation of Nrf2 Signaling and Suppression of Snail1 Transcription Factor. Biomed. Pharmacother. 141, 111870. 10.1016/j.biopha.2021.111870 34246192

[B28] Khitan Z. Harsh M. Sodhi K. Shapiro J. I. Abraham N. G. (20142014). HO-1 Upregulation Attenuates Adipocyte Dysfunction, Obesity, and Isoprostane Levels in Mice Fed High Fructose Diets. J. Nutr. Metab. 2014, 1–13. 10.1155/2014/980547 PMC4175747 25295182

[B29] Kinderlerer A. R. Pombo Gregoire I. Hamdulay S. S. Ali F. Steinberg R. Silva G. (2009). Heme Oxygenase-1 Expression Enhances Vascular Endothelial Resistance to Complement-Mediated Injury through Induction of Decay-Accelerating Factor: a Role for Increased Bilirubin and Ferritin. Blood 113 (7), 1598–1607. 10.1182/blood-2008-04-152934 19036700

[B30] Lamalice L. Le Boeuf F. Huot J. (2007). Endothelial Cell Migration during Angiogenesis. Circ. Res. 100 (6), 782–794. 10.1161/01.RES.0000259593.07661.1e 17395884

[B31] Li M. Chiu J.-F. Kelsen A. Lu S. C. Fukagawa N. K. (2009). Identification and Characterization of an Nrf2-Mediated ARE Upstream of the Rat Glutamate Cysteine Ligase Catalytic Subunit Gene (GCLC). J. Cel. Biochem. 107 (5), 944–954. 10.1002/jcb.22197 19459163

[B32] Li R. Li X. Wu H. Yang Z. Fei L. Zhu J. (2019). Theaflavin Attenuates Cerebral Ischemia/reperfusion Injury by Abolishing miRNA-128-3p-mediated Nrf2 I-nhibition and R-educing O-xidative S-tress. Mol. Med. Rep. 20 (6), 4893–4904. 10.3892/mmr.2019.10755 31638230 PMC6854549

[B33] Li T. Mo H. Chen W. Li L. Xiao Y. Zhang J. (2017). Role of the PI3K-Akt Signaling Pathway in the Pathogenesis of Polycystic Ovary Syndrome. Reprod. Sci. 24 (5), 646–655. 10.1177/1933719116667606 27613818

[B34] Li Z. Zhu J. Wan Z. Li G. Chen L. Guo Y. (2021). Theaflavin Ameliorates Renal Ischemia/reperfusion Injury by Activating the Nrf2 Signalling Pathway *In Vivo* and *In Vitro* . Biomed. Pharmacother. 134, 111097. 10.1016/j.biopha.2020.111097 33341051

[B35] Liu S. Tian L. Chai G. Wen B. Wang B. (2018). Targeting Heme Oxygenase-1 by Quercetin Ameliorates Alcohol-Induced Acute Liver Injury via Inhibiting NLRP3 Inflammasome Activation. Food Funct. 9 (8), 4184–4193. 10.1039/c8fo00650d 29993075

[B36] Liu Y. Zhao X. Zhao C. Zhang H. Zhao Y. (2019). Responsive Porous Microcarriers with Controllable Oxygen Delivery for Wound Healing. Small 15 (21), 1901254. 10.1002/smll.201901254 30997747

[B37] Luo Z. Hu Z. Bian Y. Su W. Li X. Li S. (2020). Scutellarin Attenuates the IL-1β-Induced Inflammation in Mouse Chondrocytes and Prevents Osteoarthritic Progression. Front. Pharmacol. 11, 107. 10.3389/fphar.2020.00107 32161544 PMC7054241

[B38] Lv R. Du L. Zhang L. Zhang Z. (2019). Polydatin Attenuates Spinal Cord Injury in Rats by Inhibiting Oxidative Stress and Microglia Apoptosis via Nrf2/HO-1 Pathway. Life Sci. 217, 119–127. 10.1016/j.lfs.2018.11.053 30481506

[B39] Ma Q. (2013). Role of Nrf2 in Oxidative Stress and Toxicity. Annu. Rev. Pharmacol. Toxicol. 53, 401–426. 10.1146/annurev-pharmtox-011112-140320 23294312 PMC4680839

[B40] Munoz-Chaupuli R. Quesada A. R. Angel Medina M. (2004). Angiogenesis and Signal Transduction in Endothelial Cells. Cmls, Cel. Mol. Life Sci. 61 (17), 2224–2243. 10.1007/s00018-004-4070-7 PMC11138771 15338053

[B41] Nezu M. Suzuki N. Yamamoto M. (2017). Targeting the KEAP1-NRF2 System to Prevent Kidney Disease Progression. Am. J. Nephrol. 45 (6), 473–483. 10.1159/000475890 28502971

[B42] Park S.-A. Lee M.-H. Na H.-K. Surh Y.-J. (2017). 4-Hydroxyestradiol Induces Mammary Epithelial Cell Transformation through Nrf2-Mediated Heme Oxygenase-1 Overexpression. Oncotarget 8 (1), 164–178. 10.18632/oncotarget.10516 27438141 PMC5352084

[B43] Ridiandries A. Tan J. Bursill C. (2018). The Role of Chemokines in Wound Healing. Ijms 19 (10), 3217. 10.3390/ijms19103217 PMC6214117 30340330

[B44] Ryu M. J. Kang K. A. Piao M. J. Kim K. C. Zheng J. Yao C. W. (2014). 7,8-Dihydroxyflavone Protects Human Keratinocytes against Oxidative Stress-Induced Cell Damage via the ERK and PI3K/Akt-Mediated Nrf2/HO-1 Signaling Pathways. Int. J. Mol. Med. 33 (4), 964–970. 10.3892/ijmm.2014.1643 24503931

[B45] Sandrim V. Coeli-Lacchini F. B. Tanus-Santos J. E. Lacchini R. Cavalli R. C. (2019). Circulating HO-1 Levels Are Not Associated with Plasma sFLT-1 and GTn HMOX1 Polymorphism in Preeclampsia. Hypertens. Pregnancy 38 (2), 73–77. 10.1080/10641955.2019.1582664 30835584

[B46] Sen C. K. Ghatak S. Gnyawali S. C. Roy S. Gordillo G. M. (2016). Cutaneous Imaging Technologies in Acute Burn and Chronic Wound Care. Plast. Reconstr. Surg. 138 (3 Suppl. l), 119s–128s. 10.1097/prs.0000000000002654 27556752 PMC5207795

[B47] Shaw P. Chattopadhyay A. (2020). Nrf2-ARE Signaling in Cellular protection: Mechanism of Action and the Regulatory Mechanisms. J. Cel Physiol 235 (4), 3119–3130. 10.1002/jcp.29219 PMC13482834 31549397

[B48] Sudan K. Vijayan V. Madyaningrana K. Gueler F. Igarashi K. Foresti R. (2019). TLR4 Activation Alters Labile Heme Levels to Regulate BACH1 and Heme Oxygenase-1 Expression in Macrophages. Free Radic. Biol. Med. 137, 131–142. 10.1016/j.freeradbiomed.2019.04.024 31026585

[B49] Suzuki T. Yamamoto M. (2015). Molecular Basis of the Keap1-Nrf2 System. Free Radic. Biol. Med. 88 (Pt B), 93–100. 10.1016/j.freeradbiomed.2015.06.006 26117331

[B50] Tang H. Wu L. Chen X. Li H. Huang B. Huang Z. (2021). Paeoniflorin Improves Functional Recovery through Repressing Neuroinflammation and Facilitating Neurogenesis in Rat Stroke Model. PeerJ 9, e10921. 10.7717/peerj.10921 34123580 PMC8166241

[B51] Tejada S. Batle J. M. Ferrer M. D. Busquets-Cortés C. Monserrat-Mesquida M. Nabavi S. M. (2019). Therapeutic Effects of Hyperbaric Oxygen in the Process of Wound Healing. Cpd 25 (15), 1682–1693. 10.2174/1381612825666190703162648 31269879

[B52] Tonnesen M. G. Feng X. Clark R. A. F. (2000). Angiogenesis in Wound Healing. J. Invest. Dermatol. Symp. Proc. 5 (1), 40–46. 10.1046/j.1087-0024.2000.00014.x 11147674

[B53] Veith A. P. Henderson K. Spencer A. Sligar A. D. Baker A. B. (2019). Therapeutic Strategies for Enhancing Angiogenesis in Wound Healing. Adv. Drug Deliv. Rev. 146, 97–125. 10.1016/j.addr.2018.09.010 30267742 PMC6435442

[B54] Velnar T. Bailey T. Smrkolj V. (2009). The Wound Healing Process: an Overview of the Cellular and Molecular Mechanisms. J. Int. Med. Res. 37 (5), 1528–1542. 10.1177/147323000903700531 19930861

[B55] Wang M. Yang D. Hu Z. Shi Y. Ma Y. Cao X. (2021). Extracorporeal Cardiac Shock Waves Therapy Improves the Function of Endothelial Progenitor Cells after Hypoxia Injury via Activating PI3K/Akt/eNOS Signal Pathway. Front. Cardiovasc. Med. 8, 747497. 10.3389/fcvm.2021.747497 34708093 PMC8542843

[B56] Wu C.-T. Deng J.-S. Huang W.-C. Shieh P.-C. Chung M.-I. Huang G.-J. (2019). Salvianolic Acid C against Acetaminophen-Induced Acute Liver Injury by Attenuating Inflammation, Oxidative Stress, and Apoptosis through Inhibition of the Keap1/Nrf2/HO-1 Signaling. Oxidative Med. Cell Longevity 2019, 1–13. 10.1155/2019/9056845 PMC6535820 31214283

[B57] Wu Y. Qiu G. Zhang H. Zhu L. Cheng G. Wang Y. (2021). Dexmedetomidine Alleviates Hepatic Ischaemia‐reperfusion Injury via the PI3K/AKT/Nrf2‐NLRP3 Pathway. J. Cell. Mol. Medi 25, 9983–9994. 10.1111/jcmm.16871 PMC8572787 34664412

[B58] Xiao Q. Piao R. Wang H. Li C. Song L. (2018). Orientin-mediated Nrf2/HO-1 Signal Alleviates H2O2-Induced Oxidative Damage via Induction of JNK and PI3K/AKT Activation. Int. J. Biol. Macromolecules 118 (Pt A), 747–755. 10.1016/j.ijbiomac.2018.06.130 29959995

[B59] Xu X.-X. Zheng G. Tang S.-K. Liu H.-X. Hu Y.-Z. Shang P. (2021). Theaflavin Protects Chondrocytes against Apoptosis and Senescence via Regulating Nrf2 and Ameliorates Murine Osteoarthritis. Food Funct. 12 (4), 1590–1602. 10.1039/d0fo02038a 33471008

[B60] Zhang H. Yuan B. Huang H. Qu S. Yang S. Zeng Z. (2018a). Gastrodin Induced HO-1 and Nrf2 Up-Regulation to Alleviate H2O2-Induced Oxidative Stress in Mouse Liver Sinusoidal Endothelial Cells through P38 MAPK Phosphorylation. Braz. J. Med. Biol. Res. 51 (10), e7439. 10.1590/1414-431x20187439 30156611 PMC6110350

[B61] Zhang R. Li H. Zhang S. He H. Zhangu T.-C. Ma W. (2018b). RXRα Provokes Tumor Suppression through P53/p21/p16 and PI3K-AKT Signaling Pathways during Stem Cell Differentiation and in Cancer Cellss. Cell Death Dis 9 (5), 532. 10.1038/s41419-018-0610-1 29748541 PMC5945609

